# Novel Arenavirus Infection in Humans, United States

**DOI:** 10.3201/eid1708.110285

**Published:** 2011-08

**Authors:** Mary Louise Milazzo, Grant L. Campbell, Charles F. Fulhorst

**Affiliations:** Author affiliations: University of Texas Medical Branch, Galveston, Texas, USA (M.L. Milazzo, C.F. Fulhorst);; Centers for Disease Control and Prevention, Fort Collins, Colorado, USA (G.L. Campbell)

**Keywords:** Arenavirus, Bear Canyon virus, Tamiami virus, Whitewater Arroyo virus, lymphocytic choriomeningitis virus, viruses, United States, research

## Abstract

Immunoglobulin G against Whitewater Arroyo virus or lymphocytic choriomeningitis virus was found in 41 (3.5%) of 1,185 persons in the United States who had acute central nervous system disease or undifferentiated febrile illnesses. The results of analyses of antibody titers in paired serum samples suggest that a North American Tacaribe serocomplex virus was the causative agent of the illnesses in 2 persons and that lymphocytic choriomeningitis virus was the causative agent of the illnesses in 3 other antibody-positive persons in this study. The results of this study suggest that Tacaribe serocomplex viruses native to North America, as well as lymphocytic choriomeningitis virus, are causative agents of human disease in the United States.

## Abstract 2

TOC Summary: North American Tacaribe serocomplex viruses cause acute central nervous system disease or undifferentiated febrile illnesses.

The arenaviruses (family *Arenaviridae*, genus *Arenavirus*) known to occur in North America include Whitewater Arroyo virus (WWAV), 7 other members of the Tacaribe serocomplex (Table 1), and lymphocytic choriomeningitis virus (LCMV, the prototypic member of the lymphocytic choriomeningitis–Lassa serocomplex). Specific members of the order Rodentia are the principal hosts of the arenaviruses, for which natural host relationships have been well characterized. For example, the hispid cotton rat (*Sigmodon hispidus*) in Florida is the principal host of Tamiami virus (*6**,**7*), and the ubiquitous house mouse (*Mus musculus*) is the principal host of LCMV (*9*).

**Table 1:** Natural hosts and geographic distribution of the North American Tacaribe serocomplex viruses

Virus	Natural host(s)	Location	Reference
Bear Canyon	Large-eared woodrat (*Neotoma macrotis*), California mouse (*Peromyscus californicus*)	California, USA	(*1*)
Big Brushy Tank	White-throated woodrat (*N. albigula*)	Arizona, USA	(*2*)
Catarina	Southern plains woodrat (*N. micropus*)	Texas, USA	(*3*)
Rio Catorce	White-toothed woodrat (*N. leucodon*)	San Luis Potosí, Mexico	(*4*)
Skinner Tank	Mexican woodrat (*N. mexicana*)	Arizona, USA	(*5*)
Tamiami	Hispid cotton rat (*Sigmodon hispidus*)	Florida, USA	(*6**,**7*)
Tonto Creek	White-throated woodrat (*N. albigula*)	Arizona, USA	(*2*)
Whitewater Arroyo	White-throated woodrat (*N. albigula*)	New Mexico, USA	(*8*)

Five South American members of the Tacaribe serocomplex, LCMV, and Lassa virus are etiologic agents of severe febrile illnesses in humans (*10**,**11*). The human health significance of the North American Tacaribe serocomplex viruses has not been rigorously investigated (*12*).

Studies since the mid-1990s have shown that Tacaribe serocomplex viruses are widely distributed in the United States and Mexico and that woodrats (*Neotoma* spp.) and other members of the family Cricetidae are natural hosts of these viruses (*1**–**5**,**8**,**13**,**14*). The purpose of this study was to investigate whether humans have been infected with North American Tacaribe serocomplex viruses.

## Materials and Methods

Samples of serum (n = 1,305), plasma (n = 2), and cerebrospinal fluid (n = 70) from 1,185 persons in the United States with acute central nervous system disease or undifferentiated febrile illnesses were tested for immunoglobulin (Ig) G against the WWAV prototype strain AV 9310135 and LCMV strain Armstrong by using an ELISA as described (*15*). The samples were diagnostic specimens submitted to the Arbovirus Diseases Branch, Division of Vector-Borne Infectious Diseases, Centers for Disease Control and Prevention (CDC) (Fort Collins, CO, USA) during 1989–2000 by public health laboratories in the United States. The samples had been tested selectively by CDC laboratorians for evidence of infection with St. Louis encephalitis virus, western equine encephalomyelitis virus, and other arthropod-borne agents of human disease. These tests had not yielded a specific diagnosis for any of the cases in this study.

Information about each case was limited to patient age, sex, date of illness onset, and state from which the samples were submitted. Most (634 [53.5%]) of the 1,185 case-patients were male. Ages at illness onset ranged from 0.2 months to 93 years (median 35 years), and 982 (82.0%) of the case-patients were >10 years of age at illness onset. The period between illness onset and sample collection ranged from 0 days to 10.1 years (median 31 days). At least 1 sample from each of 580 case-patients was collected before the end of week 4 of illness; for 108 case-patients multiple samples, representing different time points, were available. Cases were geographically distributed as follows: New England, 72 cases; Mid-Atlantic, 50; South Atlantic, 141; East North Central, 96; West North Central, 73; East South Central, 78; West South Central, 42; Mountain, 177; Pacific, 96; and unknown, 360.

A 1:80 dilution and 1:320 dilution of each sample was tested against the WWAV antigen, LCMV antigen, and corresponding comparison (negative-control) antigens. The adjusted optical density (AOD) of a sample-antigen reaction was the optical density of the well coated with the test antigen minus the optical density of the well coated with the corresponding control antigen. A sample was considered positive if the AOD at 1:80 was >0.250, the AOD at 1:320 was >0.250, and the sum of the AOD at 1:80 and AOD at 1:320 was >0.750. Endpoint titers against each antigen were measured in the positive samples by using serial 2-fold dilutions from 1:320 through 1:40,960. The antibody titer of a positive sample was the reciprocal of the highest dilution for which the AOD was >0.250. Titers <320 were 160 in comparisons of titers to WWAV and LCMV in individual samples. The apparent homologous virus in an antibody-positive sample was the virus associated with the highest titer if the absolute value of the difference between the titers to WWAV and LCMV was >4-fold.

## Results

We detected antibody against an arenavirus in 41 (3.5%) of the 1,185 case-patients. Of the antibody-positive case-patients, most (27 [65.9%]) were male. Ages ranged from 4 years to 85 years (median 39 years). Antibody-positive samples were submitted from Florida, Massachusetts, and Wyoming (3 samples each) and Arizona, Idaho, Kansas, Maryland, Michigan New Mexico, New York, North Carolina, Ohio, Rhode Island, Tennessee, Washington, and Wisconsin (1 sample each). For 19 samples, state of submission was unknown.

Twelve persons had positive test results for WWAV but not LCMV; 28 for LCMV but not WWAV; and 1 for WWAV and LCMV (Table 2). In the positive samples, endpoint titers against WWAV and LCMV ranged from <320 to 10,240 and from <320 to 20,480, respectively. The apparent homologous virus was WWAV in 10, LCMV in 24, and indeterminate in 7 of antibody-positive persons (Table 2).

**Table 2:** Antibody (immunoglobulin G) titers against WWAV and LCMV in 1,185 cases of acute central nervous system disease or undifferentiated febrile illnesses, United States*

No. cases	Antibody titer		Apparent homologous virus
	WWAV	LCMV	
5	640	<320	WWAV
1	1,280	<320	WWAV
3	2,560	<320	WWAV
1	10,240	<320	WWAV
7	<320	640	LCMV
3	<320	1,280	LCMV
5	<320	2,560	LCMV
4	<320	5,120	LCMV
2	<320	10,240	LCMV
3	<320	20, 480	LCMV
2	320	<320	Indeterminate
1	640	1,280	Indeterminate
4	<320	320	Indeterminate
1,144	<320	<320	None

*WWAV, Whitewater Arroyo virus; LCMV, lymphocytic choriomeningitis virus.

Ages of the 10 persons in whom WWAV was the apparent homologous virus ranged from 5 to 70 years (median 43 years). Samples from these persons were submitted from Arizona, New Mexico, and North Carolina (1 sample each) and Florida and Wyoming (2 samples each); for 3 samples, state of submission was unknown.

The ELISA included paired samples from 8 antibody-positive persons. Time from onset of illness to the first samples from these persons ranged from 0 to 47 days. In side-by-side tests, the endpoint titer to WWAV in the second sample was >4-fold higher than that to WWAV in the first sample in paired samples from 2 persons, and the endpoint titer to LCMV in the second sample was >4-fold higher than that to LCMV in the first sample in paired samples from 3 of the 6 other antibody-positive persons (Table 3).

**Table 3:** Antibody (immunoglobulin G) against WWAV and LCMV in paired serum samples from humans with acute central nervous system disease or undifferentiated febrile illnesses, United States*

Case-patient no.	Age, y, at illness onset	Days after illness onset			Antibody titer, WWAV			Antibody titer, LCMV		Apparent homologous virus
S1	S2	S1	S2	S1	S2
1	32	14	44		<320	640		<320	<320	WWAV
2	65	15	61		<320	2,560		<320	<320	WWAV
3	38	14	33		<320	<320		5,120	5,120	LCMV
4	51	2	68		<320	<320		320	20,480	LCMV
5	59	24	38		<320	<320		320	5,120	LCMV
6	72	0	15		<320	<320		<320	640	LCMV
7	12	25	33		<320	<320		320	320	Indeterminate
8	25	47	123		<320	<320		320	320	Indeterminate

*WWAV, Whitewater Arroyo virus; LCMV, lymphocytic choriomeningitis virus; S1, first sample; S2, second (last) sample in paired samples.

## Discussion

Previously, antibody to Tamiami virus was found in 5 (3.8%) of 131 Seminole Indians sampled in southern Florida (*16*), and antibody to a Tacaribe serocomplex virus was found in 2 (0.24%) of 829 persons who had worked with cricetid rodents in North America (*15**,**17*). The results of our current study strengthen the notion that Tacaribe serocomplex viruses enzootic in North America are infectious in humans. The increase in antibody titer against WWAV in cases 1 and 2 in this study (Table 3) suggests that a North American Tacaribe serocomplex virus caused the illnesses in these persons.

The WWAV strain AV 9310135 was originally isolated from a white-throated woodrat (*N. albigula*) captured in northwestern New Mexico (*8*). A recent study demonstrated a high level of diversity among the amino acid sequences of the structural proteins of the North American Tacaribe serocomplex viruses (*5*). Hypothetically, human IgG against some North American Tacaribe serocomplex viruses is not strongly reactive against WWAV in ELISA. If so, the prevalence of antibody to Tacaribe serocomplex viruses in this study actually might be >3.5%.

The severity of human disease caused by LCMV ranges from mild febrile illness to severe encephalitis and disseminated disease (*18*). The results of this study suggest that the illnesses in case-patients 4–6 (Table 3) were caused by LCMV. Whether samples from these 3 persons were tested for anti-LCMV antibody (IgM or IgG) by clinical laboratories could not be determined from records maintained at CDC.

Specimens from 33 of the antibody-positive persons in this study were limited to single specimens. Perhaps these illnesses were caused by a North American Tacaribe serocomplex or by LCMV. The antibody titer to WWAV in the antibody-positive person from New Mexico was 10,240 in a serum sample collected on day 22 day after illness onset.

Future studies on the relevance to human health of the North American Tacaribe serocomplex viruses should include defining the clinical spectrum and epidemiology of human disease caused by these viruses. Some of these viruses may cause aseptic meningitis, encephalitis, or meningoencephalitis.Thus,humandiseasecausedbyNorthAmericanTacaribeserocomplexvirusesmay be confused with severe encephalitis caused by LCMV, especially in persons who report recent exposure to rodents.
